# Genome-wide estimation of gender differences in the gene expression of human livers: Statistical design and analysis

**DOI:** 10.1186/1471-2105-6-S2-S13

**Published:** 2005-07-15

**Authors:** Robert R Delongchamp, Cruz Velasco, Stacey Dial, Angela J Harris

**Affiliations:** 1 Division of Biometry and Risk Assessment, National Center for Toxicological Research, Jefferson, Arkansas 72079; 2 School of Public Health, Louisiana State University Health Sciences Center, New Orleans, Louisiana 70112; 3 Center for Hepatotoxicity, National Center for Toxicological Research, Jefferson, Arkansas 72079; 4 Center for Toxicology and Environmental Health, LLC, 615 W. Markham, Little Rock, Arkansas 72201

## Abstract

**Background:**

Gender differences in gene expression were estimated in liver samples from 9 males and 9 females. The study tested 31,110 genes for a gender difference using a design that adjusted for sources of variation associated with cDNA arrays, normalization, hybridizations and processing conditions.

**Results:**

The genes were split into 2,800 that were clearly expressed (expressed genes) and 28,310 that had expression levels in the background range (not expressed genes). The distribution of p-values from the 'not expressed' group was consistent with no gender differences. The distribution of p-values from the 'expressed' group suggested that 8 % of these genes differed by gender, but the estimated fold-changes (expression in males / expression in females) were small. The largest observed fold-change was 1.55. The 95 % confidence bounds on the estimated fold-changes were less than 1.4 fold for 79.3 %, and few (1.1%) exceed 2-fold.

**Conclusion:**

Observed gender differences in gene expression were small. When selecting genes with gender differences based upon their p-values, false discovery rates exceed 80 % for any set of genes, essentially making it impossible to identify any specific genes with a gender difference.

## Background

Liver toxicity is the most common adverse event associated with the introduction of a new drug despite extensive pre-clinical toxicity testing. The failure to predict this toxicity is attributed to differences among species in the metabolism and disposition of certain chemicals and drugs. This spawns an interest in *in vitro *tests that use human hepatocytes. The scarcity of primary human hepatocytes and the unsuitability of many human cell lines, which were derived from liver cancer cells, are a serious limitation for developing such tests. In addition, primary hepatocytes differentiate quickly in culture, restricting their use to short term studies [1]. The National Center for Toxicological Research (NCTR) embarked upon a program to develop conditionally immortalized cell lines as potential *in vitro *models to evaluate the liver toxicity of new drugs [2,3]. A further incentive for this approach is the potential to study mechanisms of liver toxicity from different genders and/or ethnic populations. As a start and proof of principle, a study was proposed to develop and characterize conditionally immortalized human primary hepatocyte cell lines from female and male donors.

It is desirable to quantify in some way that an immortalized cell line retains functions characteristic of primary hepatocytes. Since cDNA arrays can screen thousands of genes for expression differences, they are attractive as an evaluation tool. Once some immortalized cell lines are characterized with respect to primary hepatocytes, cDNA arrays could monitor the persistence of gender differences in expression across these immortalized cell lines, thereby showing retention of some functions without using primary hepatocytes. Toward these ends, this study scanned the genome for expression differences in the livers between 9 males and 9 females.

The use of cDNA arrays to assay for gender difference encounters two statistical problems that are not simple to deal with. First, gender comparisons relying upon array technologies are subject to biological and technical sources of variation [4]. The experimental design needs to avoid confounding technical variation within treatments. This study modified an experimental design, which was previously employed for two-dye per array hybridizations [5], to ^33^P-labeled filter arrays that are stripped and reused. Second, there is a potential for excessive false positive rates because of the number of genes evaluated. For our purposes, a set of genes is needed for which the false positive rate is acceptably low. The false positive rates associated with potential sets were evaluated using recently developed *post hoc *methods based upon the empirical distribution of the observed p-values [6,7].

## Methods

### Human Liver

Segments of human liver were obtained from Dr. Fred Kadlubar, Division of Molecular Epidemiology, NCTR (Jefferson, AR) and Dr. Steven Strom, the University of Pittsburgh (Pittsburgh, PA). This project was approved by the Research Involving Human Subjects Committee of the Food and Drug Administration (FDA). Nine pairs, a male paired with a female, were formed from available subjects. Pairs were processed concurrently to control variation from technical sources associated with sample preparation and measurement, and this was the major reason for pairing subjects. In forming pairs, we also attempted to match the age, race, and smoking/drinking habits of subjects as much as possible. However, this matching was not rigorous. The age of each subject was known (range: 25–58). For some subjects, information concerning their race (Caucasian and Hispanic) and their smoking and/or drinking habits was available although this information was not complete. In addition, several of these subjects are known to have died in a hospital where they were administered drugs in a failed attempt to stabilize their condition.

### RNA Isolation

Total RNA was isolated from each liver sample using TRIzol (Life Technologies, Rockville, MD) according to the manufacturer's recommendations. Purified RNA was then treated with DNAse to remove residual DNA contamination. One tenth volume of 10X DNAse buffer (0.4 M Tris-HCl, pH 7.9; 0.1 M NaCl, 60 mM MgCl2, 1.0 mM CaCl2) was added to a reaction containing 50 to 100 μg of purified RNA and one unit RQ1 RNAse-free DNAse (Promega, Madison, WI). The reaction was then incubated for 15 min at 37°C, extracted once with an equal volume of phenol/chloroform, precipitated with ethanol and finally resuspended in RNAse-free dH_2_O. RNA yields were determined by spectrophotometric analysis. RNA integrity was confirmed by gel electrophoresis.

### Filter Information

The filters used in this experiment were GF200, GF201, GF203, GF204, GF205 and GF206 (Invitrogen, Carlsbad, CA). Each filter was spotted with 5,184 expressed sequence tags (ESTs) representing human genes.

### Filter Array Analysis

Pre-wetted filters were prehybridized at 42°C for 2 hr in 0.75 M NaCl, 0.17 M NaPO_4 _buffer (pH 7.0), 0.15 M Na_4_P_2_O_7 _• 10 H_2_O, 5X Denhardt's solution, 2.0% SDS, 100 μg/ml denatured salmon sperm DNA, 50% formamide and 5.0 μg human Cot-1 DNA. Five micrograms of total RNA were combined with 2.0 μl of 1.0 μg/ml oligo dT primer (LifeTechnologies) in a total volume of 10.0 μl and then incubated for 10 min at 70°C. The reaction was quick-chilled on ice for 2 min. The following components were added to the oligo dT-primed RNA; 6.0 μl of 5X first strand buffer (Clontech, Palo Alto, CA), 1.5 μl of 20 mM dNTPs (dGTP, dTTP, dCTP) (Invitrogen), 1.0 μl of 0.1 M DTT, 1.5 μl of PowerScript reverse transcriptase (RT) (Clontech) and 10 μl of α-^33^P dATP (>3000 Ci/mmol) (ICN, Irvine, CA). The reaction was incubated for 90 min at 42°C. Unincorporated nucleotides were removed by column purification using Bio-spin 6 columns (Biorad, Hercules, CA). Incorporation of label for all targets ranged ± 20% from the mean. The radiolabeled target was denatured by boiling for 3 min and added to 5 ml of prehybridization solution. The filters were hybridized with the denatured target for 18–20 hr at 42°C. After hybridization, the filters were washed twice in 2X SSC, 1% SDS at 68°C for 30 min and twice in 0.5X SSC, 0.5% SDS at 68°C for 30 min

### Data Imaging

The washed, hybridized filters were sealed in plastic sheet protectors and exposed on a Molecular Dynamics phosphor-screen (Amersham, Piscataway, NJ) for 72 hr. The screens were imaged with a Storm Phosphorimager (Amersham) at a resolution of 50 microns. The median pixel intensity for each spot was determined using ArrayVision software (Research Imagine, Ontario, Canada). Each filter was stripped after imaging as recommended by the manufacturer. Briefly, boiling 0.5% SDS was poured into a large glass dish. Hybridized filters were placed in the hot solution and agitated for one hour without additional heating. The filters were then imaged for four hr on a phosphor screen (Amersham Biosciences) at a resolution of 200 microns. Each filter was stripped five times.

### Statistical Design

This study examined gene expression in liver tissue from 9 male subjects and 9 female subjects. Each male was paired with a female during the assay of gene expression in an effort to control technical variation associated with arrays, hybridizations and processing conditions. Each liver sample was hybridized to two arrays. First, one of the pair was assigned to an array, and the other sample was assigned to the other array. After the initial hybridization, both arrays were stripped and the array assignments of the samples were swapped for the second hybridization. These four expression measurements form a 'block'. Intensities were recorded at development times of 16 and 72 hours yielding 8 observed intensities for a block. The 9 blocks yielded 72 intensities per interrogated spot. With this design, we estimate the effects of blocks, effects of arrays within blocks, effects of hybridization (first or second) within blocks, and effects of subjects within blocks (Table 1). The pair of samples, which form a block, was processed concurrently. Thereby, variation associated with conditions and reagents involved in mRNA extraction, reverse transcription, hybridization, and washing steps are presumed to be smaller within blocks. This design mimics the simultaneous hybridization of two-dye platforms by taking advantage of the capability for membrane arrays to be reused. This allows the estimation of an array effect for each spot, which has been recognized as one of the largest contributors to intensity variability in the radio-labeled platform [8].

### Analysis of Covariance

An analysis of covariance was fit to the log-intensity data for each spot. No background correction was applied. This model estimated the difference between the male subject's log-intensities and the female subject's log-intensities, i.e., this analysis produced an estimate of the sex difference in every block. These 9 estimates are adjusted for the factors in the experimental design and they are normalized by the median. In addition, a similarly adjusted and normalized average magnitude of the log-intensities was estimated.

Table 1 is a typical analysis of variance table for the statistical model that was fit to each spot. The median of all the spot intensities that were observed at each array-hybridization-time was first computed. These medians were entered as covariates to normalize the log-intensities [9] and their effect in the model is similar to a global normalization. The least squares estimate of the difference in log-intensities between the male sample and female sample was computed for each block. These estimates are normalized by the median and they are also adjusted for the main effects of time, block, array, and hybridization. An 'average adjusted log-intensity' was also computed for each spot. These estimates are the least squares means evaluated at 'median = 10' and the average levels of the categorical factors. This analysis was implemented using 'PROC GLM' [10].

### Loess Regression

Within each array type (5352 spots) and block, the estimates of the gender difference were plotted against their estimate of the average magnitude, e.g., Figure 2. Observed trends were removed by Loess regression using 'PROC LOESS' [10]. This program allows one to specify several parameters that govern the degree of smoothing. Herein, we selected a quadratic equation, a bandwidth of 10% of the data (about 500 observations), and applied the smoothing algorithm three times to mitigate the influence of 'outliers'. Other combinations of these smoothing parameters were also tried, and they did yield differences in details, which we elaborated in the Discussion.

### Selecting Expressed Genes

The adjusted average log-intensity for many of the interrogated genes evidenced little if any expression. Genes that are not expressed cannot be differentially expressed. So, it is useful to separate "expressed" genes from "not expressed" genes in analyses. Genes were partitioned into "expressed" and "not expressed" groups based on their adjusted average log-intensity. Essentially, high intensities are unlikely to have resulted from cross hybridization or other background sources, while low intensities are likely to represent a substantial amount of cross hybridization. The empirical distribution of the adjusted average log-intensity estimates was examined in a normal probability plot to determine a reasonable cut point. In liver samples that do not contain any mRNA matching a spotted cDNA sequence, i.e., a gene that is not expressed, the observed hybridization log-intensity is a background level arising from cross hybridization plus measurement error. When a large number of genes are not expressed in all of the liver samples, their intensities being of similar magnitude produce an obvious mode at the low end of the empirical distribution. Values less than this mode are assumed to arise entirely from "not expressed" genes. We also assumed that the distribution is symmetric about this mode and approximated this component of the empirical distribution by a normal distribution [11], which can be estimated directly from the normal probability plot. The genes were partitioned into "expressed" genes and "not expressed" genes based on a cutoff, which gives a low probability that larger values arise from the normal distribution. Genes with values greater than the cutoff were classified as "expressed" and the rest were classified as "not expressed".

### Selecting Genes with Gender Differences in Expression

A few genes were spotted more than once. The study examined 32,112 spots representing 31,110 genes (distinct Gene Bank accession numbers). The analysis of covariance/Loess regression generated 9 smoothed estimates of log-fold changes for each spot. Estimates from replicated spots were averaged so that there was one estimate per block and gene. Likewise, replicated estimates of the adjusted average log-intensities were averaged. This resulted in 9 estimates of the gender difference and an estimate of the average magnitude for each interrogated gene.

The smoothed estimated log-fold changes were averaged  and their standard error  was computed. Two-sided p-values and 95 % confidence bounds were calculated on the assumption that  has a t-distribution with 8 degrees of freedom. We also computed bootstrap samples under the assumption of symmetry under the null hypothesis. Essentially, the bootstrap p-values duplicated those from the t-distribution and they were not reported.

There were 31,110 tests of the hypothesis that there was no gender effect. Simply selecting genes where the p-values are less that 0.05 would lead to an excessive number of false positives. Our strategy for dealing with the false-positive problem is elaborated elsewhere [7]. P-values order genes according to the evidence for the null hypothesis. Genes having gender differences in expression are more likely to have small p-values and this is seen in a departure of their empirical distribution from its uniform expectation under the null [7,12]. Herein, the observed distribution was assumed to be a mixture distribution with a proportion of the values having a uniform distribution, i.e., no gender difference in the expression, and the remainder having a Beta distribution, i.e., sexes differed. The mixing proportion and Beta parameters were estimated by maximizing the likelihood of this mixture distribution [6]. The estimated mixture components were used to estimate false discovery rates for subsets of genes classified as 'having a gender difference' because their p-value is less than a specified value [7].

## Results

### Data Completeness

This study interrogated 32,112 cDNA spots using six types of arrays, each having 5352 spots. For each spot, the data are typically 72 observations, i.e. log-intensities from 9 blocks × 2 arrays × 2 hybridizations (hyb) × 2 development times. With no missing values, there would be 32,112 × 72 (2,312,064) observations. About 2 % of the data was discarded because the quality of the image from the phosphorimager was judged to be unsatisfactory, 48,168 observations: data from 6 of the 16 hr development times and 3 of the 72 hr development times. All block-array-hybridization combinations have data from at least one development time and all blocks have at least 7 observations out of the 8 that were planned for. Thus, gender differences were estimable for all blocks and genes.

Scatter plots of the accepted data suggest that there may be a few outlier values on some arrays. We did not attempt to remove 'outliers'. Most occurred in spots with background levels of expression and any apparent gender differences in these genes were classified as false positives because the gene was essentially 'not expressed' in this study. The few wider confidence bounds seen in Figure 6 may represent a contribution from an outlier.

### Analyses of Covariance

**Figure 1:**
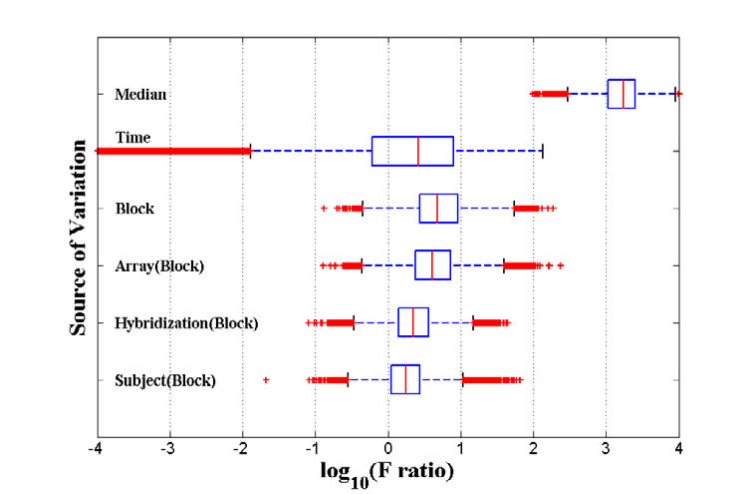
**Box plots summarizing the logarithm of F-ratios computed in the 32,112 analyses of covariance. **The F-ratios for six sources of variation are each represented by a box plot. The box encloses the range between the first and third quartile. The interior line is the median (Table 1). The dashed blue line encloses the interior 95% of the observations. More extreme values are plotted with a plus (+) symbol; a few values for 'Time' less than -4 and values for 'Median' greater than 4 are not shown.

The data for a spot were partitioned into six sources of variation as outlined in Table 1. The residual variation is the sum of squared differences between the 16 hour and 72 hour intensities after adjusting for 'median' and 'time'. This source largely measures variation associated with aligning the data-capture template with the actual cDNA spots and the process of counting radioactive decays. In particular, it does not have any variation due to subject differences, array differences, sample preparation differences or hybridization differences (Expected mean squared errors, Table 1). Conceptually, the residual is a lower bound on measurement errors. The ratio of the respective mean squared errors with the residual mean squared error (F-ratio, Table 1) give the relative magnitudes of variation as partitioned in this study. Table 1 reports the median of the 32,112 F-ratios for each source along with the p-value for an F-ratio with the table's degrees of freedom. Figure 1 summarizes these ratios from all spots as box plots. The dominant source of variation is associated with the regression using the median as a covariate. This is the median of the log-intensities of the 5,352 spots that were interrogated on an array. The log_10_(F ratio) is essentially greater than 2 for all spots with a median value of 3.23. The remaining sources generally have log_10_(F ratio)s that exceed 0 but usually do not exceed 1. Because the EMS for the source, 'Blocks', includes variance components from arrays, hybridizations, and subjects, the F-ratios for 'Blocks' should be larger than those for 'Array(Block)', 'Hyb(Block)', or 'Sex(Block)', and this is the case in Figure 1. The smallest source of variation is 'Sex(Block)', median log_10_(F ratio): 0.24. Since the box plot for 'Sex(Block)' is not centered over 0, this source usually exceeds the residual variance implying that . However, this source is not statistically significant for most spots implying that the variation among subjects and any sex effects are small for most of the spots. The median F ratio of 10^0.24 ^= 1.74 with 9 and 34 degrees of freedom has a p-value of 0.12.

In the analysis of covariance, least squares estimates of the logarithm (base 2) of the fold-change in gene expression, males/females, were computed for each block and spot. These are hereafter referred to as "estimated log-fold changes". Likewise, the least squares estimates of the expected log-intensity evaluated at median = 10 and the mean levels of the other factors, i.e., block, time, hyb, and array, were computed for each spot. We refer to these estimates as "adjusted average log-intensities".

### Adjustment by Loess regression

**Figure 2:**
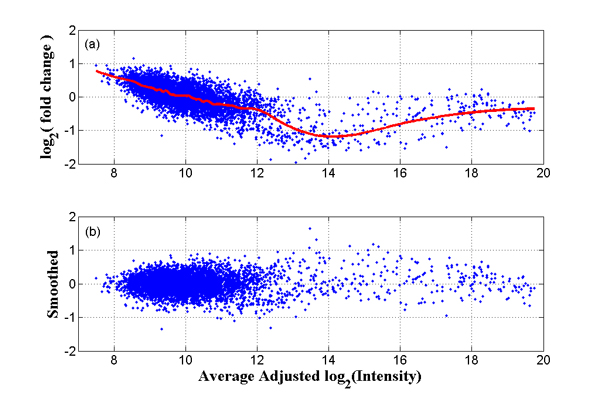
**Scatter plot of estimated fold-changes before (a) and after (b) Loess smoothing. **Figure 2a plots 5,352 estimates of the gender difference in expression from the analyses of covariance. These estimates are for 'Block 5' and the genes interrogated on array type, GF201. The red line is the trend estimated by Loess regression. Figure 2b plots these data after removing the trend.

The upper panel of Figure 2 plots estimated log-fold changes from block 5 and array type GF201 (5352 spots) against the adjusted average log-intensities. This figure is representative of trends observed over blocks and array types in the sense that the estimates exhibit systematic deviations from a horizontal line at 0 and these deviations tend to affect all spots within a neighborhood. The magnitude and direction of these deviations differ by block and array type. So, it is unlikely that these deviations represent gender differences. Such trends were removed by Loess regression computed within each block and array type, e.g., lower panel of Figure 2, yielding smoothed estimated log-fold changes.

### Selecting expressed genes

**Figure 3:**
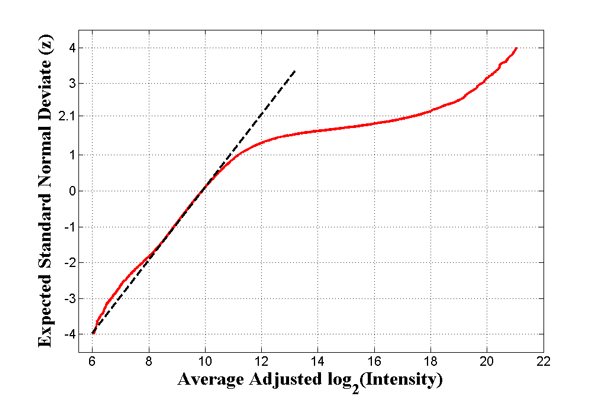
**Normal probability plot of the estimates of the adjusted average log-intensities**. The red line, actually 31,110 points, is a normal probability plot of the adjusted average log-intensities. The dashed line indicates a normal distribution that roughly approximates the lower values, and it was used to model the background distribution of log-intensities. Our interpretation was that adjusted average log-intensities exceeding 12 represent expressed genes.

The 32,112 spots represent 31,110 unique Genebank accession numbers (genes). Replicate estimates of adjusted average log-intensities were averaged to yield a single estimate for a gene. Figure 3 is a normal probability plot of the 31,110 adjusted average log-intensities. The dashed line indicates that the lower values can be approximated by a normal distribution. We assume that this distribution models the hybridization that occurs when the samples do not contain any mRNA matching the spotted cDNA sequence, i.e., genes that are not expressed in the sample. Under this normal model for 'not expressed' genes, values exceeding 12 are unlikely, *Pr *[*z *> 2.1] = 0.018, to arise when a gene is not expressed in at least some of the samples. This cut point partitioned the interrogated genes into 2,800 'expressed' genes and 28,310 'not expressed' genes. Most of the genes for cytochrome P450 enzymes were classified as 'not expressed'. This probably reflects a limited ability to discern low average levels of expression from background.

### Selecting genes with gender differences in expression

**Figure 4:**
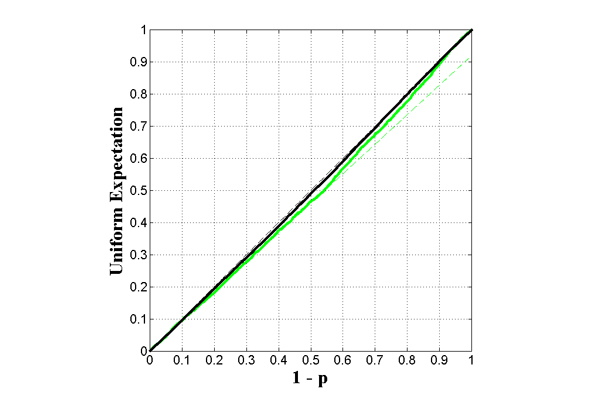
**Cumulative frequency distribution of 1-p (p-value plot) for expressed genes and 'not expressed' genes. **The distribution for expressed genes is green and the distribution for 'not expressed' genes is black. The p-values are from the t-tests of a gender difference. The dashed lines indicate a theoretical uniform distribution for p-values under the null hypothesis; the black line assumes that all of the 'not expressed' genes fit the null hypothesis and the green line assumes that 92% of expressed genes fit the null hypothesis.

A p-value assessing the evidence of 'no gender effect' was computed for each gene. Figure 4 plots these p-values (1 - p) against their expectation under a uniform distribution. In this plot, departures from the diagonal indicate that the p-values are not uniformly distributed. The p-values from the 'not expressed' genes are close to the diagonal, which corroborates our assumption that these genes cannot differ because they are not expressed. The expressed genes depart from the diagonal. The observed distribution of p-values for the expressed genes was fit with a mixture model, which estimates that 92% (2,576 genes) of the expressed genes arise from a uniform distribution, i.e., no gender difference, and 8% (224 genes) of the expressed genes have a beta (1.82, 5.89) distribution, i.e., gender difference.

**Figure 5:**
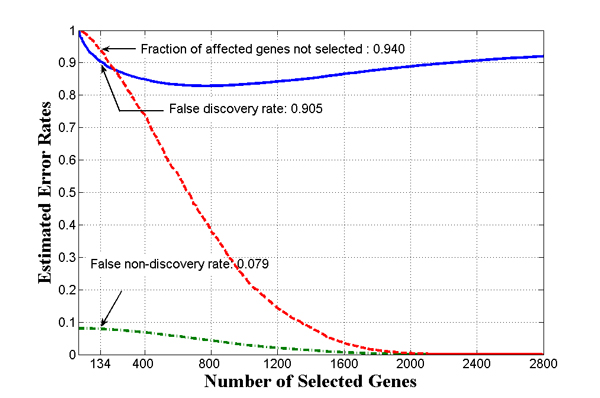
**Expected classification errors for genes that are selected based upon their p-value from the t-test for a gender difference. **When the genes are ordered by their p-values from the t-test of a gender difference, the horizontal axis indicates the set of genes selected as having a gender difference. The vertical axis plots associated estimates of the false discovery rate (solid blue line), false non-discovery rate (dash-dotted green line), and proportion of affected genes not selected (dashed red line). These estimates assume that 8% of the 2800 expressed genes, 224 genes, have a gender difference where the 8% was estimated by fitting a mixture of distributions model to the observed p-values.

Figure 5 plots expected error rates for gene selections based upon their p-values. For example, 134 genes have p-values less than 0.05. The estimated false discovery rate for these 134 genes is 0.905, which implies that the expression for 121 of these selected genes does not actually differ by gender. The estimated false non-discovery rate is 0.079. That is, 211 genes having a sex difference are expected among 2,576 genes that are not selected. The fraction not selected is 0.94, 211 out of the 224 genes that are predicted to differ between the sexes. Figure 5 indicates that the false discovery rate (solid blue line) would exceed 80% for any set of genes selected because their p-values are smaller than a specified value.

**Figure 6:**
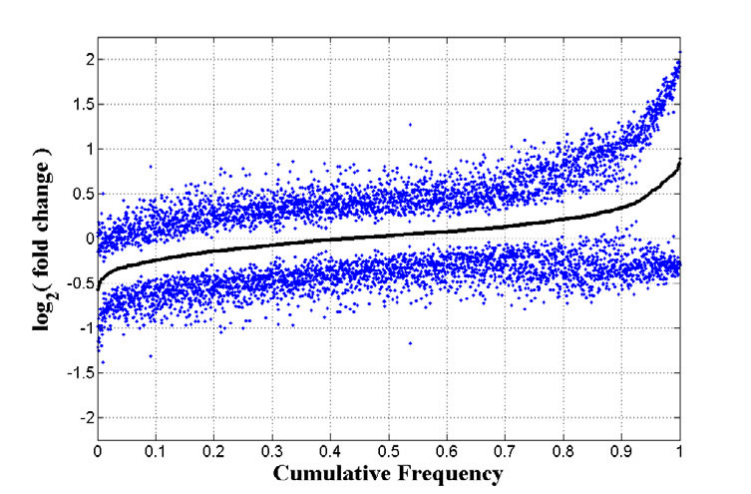
**Estimated fold-changes in gene expression of males relative to females for 2800 expressed genes**. The black points are the averages of the 9 Loess-smoothed estimates of the logarithm (base 2) of the fold change for the 2,800 expressed genes. The estimates were ordered from smallest to larges and the horizontal axis gives the cumulative proportion. Blue points are upper and lower 95 % confidence bounds.

The average of the Loess-smoothed estimates (black) and their 95% confidence bounds (blue) are plotted for the 'expressed' genes in Figure 6. The horizontal axis in this plot is the rank of the average among the 2800 'expressed' genes. All of the observed gender differences in gene expression are small. Essentially all of the point estimates (99.7 %) are within the interval, [-0.5, 0.5]. That is, observed fold changes were less than . Furthermore, 79.3 % of the 95 % confidence intervals are within the interval, [-0.5, 0.5] and most (98.9 %) are within the interval, [-1,1]. Only 27 genes have confidence intervals that are not within the interval, [-1,1]. In concept, these 27 genes could have gender differences larger than 2 fold. However, wide bounds usually reflect an outlier observation, which inflates the standard deviation.

## Discussion

This study aimed to identify genes in human livers for which expression differed between the sexes. Expression was assayed for 31,110 genes of which 2,800 were classified as "expressed". The remainder of the interrogated genes had observed expression intensities that were indistinct from background, and they are classified as "not expressed". However, most of the genes for the cytochrome P450 enzymes were in this "not expressed" subset, which suggests that detection was limited by background and/or the sensitivity of the labeled target. Evidence of a gender difference was evaluated by a t-test, and a mixture model was fit to the observed p-values from these tests (Figure 4). This model estimated that expression in 8 % [95 % CI: 7 % to 9 %] of the 2,800 "expressed" genes differed by gender. However, the ability of this study to identify specific genes is poor with estimated false discovery rates exceeding 80 % for every partition of the 2,800 genes (Figure 5).

The study estimated the relative difference in expression for all interrogated genes. These estimates were plotted for the "expressed" genes (Figure 6). All estimates are less than 1.55 fold and they generally have narrow confidence intervals. Any gender differences that might be detectable through these arrays would be small, which excludes their anticipated use to monitor the persistence of gender differences across immortalized cell lines.

The experiment was designed to adjust estimated gender differences for several sources of variation (Table 1). Since this design made twice as many measurements – two arrays per sample, this yielded more precise estimates of gender differences than would have been possible with one array per liver sample. This design was proposed because we had access to a limited number of liver samples, and arrays were relatively inexpensive. Conceptually, one could have used the same number or arrays with twice as many samples. Figure 1 showed that components of variation associated with arrays and hybridizations are somewhat larger than the variation associated with subjects. These components do not impact the variation in the estimated gender difference under the implemented design. However, they would under a one array per sample design, and any precision gained in estimating the subject component would be offset by the addition of array and hybridization components. Because these two designs would estimate the respective variances of the gender difference with different degrees of freedom, the better of the two designs would depend on the number of blocks. In all but cases with a few blocks, the implemented design would be better.

Figure 2 illustrates a case where there was a systematic trend in the least squares estimates. There were substantial trends in about half of the block – array type combinations. However, the needed correction was not similar either within an array type or within a sample block. Consequently, estimated gender differences would be much more variable without the Loess smoothing. We suspect that these trends are caused by spatial variation in binding due to intrinsic properties of membranes and/or irregular distribution of labeled solution, besides it seems unlikely that real gender differences would exhibit this behavior. Smoothing eliminates much of this variation, but it also reduces the estimated difference. We tried several smoothing levels ranging from no smoothing to very local smoothing. Figure 2 represents an intermediate level of smoothing, which was adopted for this report as a representative example. The smoothing levels that we tried gave similar distributions for the p-values, and the same general conclusions would be reached. Namely, there is evidence of a few small changes but the specific genes cannot be identified. However, the rank of a specific gene in Figure 6 or the genes included in a 'significant' set (Figure 5) depends far too much on decisions about the smoothing parameters.

The estimate that 8 % of the "expressed" genes have gender differences relies on the assumption that the p-values from genes with no gender difference in expression have a uniform distribution. Since these p-values are based on a t-test, this assumption requires that the mean gender difference for each gene have a normal distribution. This is not unreasonable on theoretical grounds. Further, bootstrap estimates are largely within the expected range if they are considered as estimates of the t-test's p-values (data not shown). That is, the assumption of a symmetric distribution under the null hypothesis gives a distribution for the mean that is well approximated by the t-distribution.

More problematic is the assumption that the 2,800 tests are independent. Correlations are induced through shared conditions by all genes on an array, the normalization step, and the Loess regression step. Further, expression levels among some genes are expected to be correlated because they work in concert to achieve a specific cellular structure or function. Simulation studies have shown that the estimated number of affected genes is not biased by correlations among tests, but correlations increase the variance of this estimate substantially [13,14]. The reported confidence bounds on the proportion of affected genes assume independence and likely underestimate the actual variation, possibly by a substantial amount. Consequently, the statistical significance of the 8 % estimate is not clear.

P-values were computed for all interrogated genes. Genes that are not expressed should not have intensities that depend on sex. The p-values for the "not expressed" subset show little departure from the diagonal (Figure 4), which is "as expected" if they arise under the null distribution. These genes also have correlations induced by shared conditions, normalization and Loess regression. Apparently, these correlations were insufficient to disrupt the uniform distribution for these p-values.

## Conclusion

We estimated that the gene expression of 224 genes differed between sexes. The observed gender differences in expression were small. False discovery rates exceed 80 % for every set of genes selected by their p-values, essentially making it impossible to identify any specific genes with a gender difference.

## Authors' contributions

RRD and CV developed the statistical design, analyzed the data, and wrote the manuscript. SD carried out the preparation of the liver samples and the cDNA array hybridizations. AJH conceived of the study, participated in its design and coordination, obtained the grant and helped to draft the manuscript. All authors read and approved the final manuscript.

## Figures and Tables

**Table 1:** Analysis of covariance model that was applied to the data for an interrogated spot. This table gives the typical degrees of freedom and the approximate expected mean squared errors for the sources of variation estimated under the experimental design. The reported F-ratios are the median of the 32,112 analyses. This median is the vertical red line in the box of Figure 1. The p-value is for the tabled F-ratio with numerator degrees of freedom as tabled and 34 denominator degrees of freedom.

**Source**	**df**	**Expected Mean Squared Error**	F ratio*	**P value**
Median	1	*σ*^2 ^+ *Q *(Time, Median)	1715.4	<.0001
Time	1	*σ*^2 ^+ *Q *(Time)	2.62	0.1145
Block	8		4.80	0.0005
Array (Block)	9		4.10	0.0012
Hyb (Block)	9		2.24	0.0437
Subject (Block)	9		1.75	0.1143
Residual	34	*σ*^2^		

* median value of the ratios, mean squared error / residual MSE, for the 32,110 spots.
